# Editorial: Last universal common ancestor and origin of life: what uncultivated Bacteria, Archaea, and extremophiles can tell us

**DOI:** 10.3389/fmicb.2024.1412625

**Published:** 2024-06-03

**Authors:** Bhagwan Narayan Rekadwad, Juan M. Gonzalez, Wen-Jun Li

**Affiliations:** ^1^MicrobeAI Lab, Division of Microbiology and Biotechnology, Yenepoya Research Centre, Yenepoya (Deemed to be University), Mangalore, India; ^2^Institute of Natural Resources and Agrobiology, Spanish National Council for Research, IRNAS-CSIC, Sevilla, Spain; ^3^State Key Laboratory of Biocontrol, Guangdong Provincial Key Laboratory of Plant Resources and Southern Marine Science and Engineering Guangdong Laboratory (Zhuhai), School of Life Sciences, Sun Yat-sen University, Guangzhou, China; ^4^State Key Laboratory of Desert and Oasis Ecology, Xinjiang Institute of Ecology and Geography, Chinese Academy of Sciences, Ürümqi, China

**Keywords:** origins of life, anammox bacteria, microbial evolution, primordial metabolism, thermodynamics, microbial dark matter

In the era of synthetic biology, quantum computing, and artificial intelligence, the last common universal ancestor (LUCA) and the origin of life remain major unanswered questions about life on Earth (Pross and Pascal, 2013). This Research Topic focused on anticipating a burning question: “Who was LUCA?” Various theories have been proposed. In particular, proposed thermodynamic theories suggest a chemical origin for extremophilic life on Earth and beyond (Szostak, 2018). Wimmer et al. noted that any reaction of this nature occurs at the expense of energy, which facilitates specific chemical reactions. Therefore, it was necessary to determine whether external energy was involved in the genesis of LUCA from existing materials. These authors (Wimmer et al.) have reported more than 400 reactions, revealing that the chemical constituents of LUCA require no external energy sources such as UV light or electrical discharge. The energy required to generate LUCA may have come from sources such as soluble, surface-catalyzed reactions, radiating geochemical energy, phosphide minerals, etc., or even from simple chemical reactions such as the breakdown of hydrogen bonds in various molecules. This means that the biosynthetic reactions that occur during the formation of LUCA may follow the normal thermodynamic path of the metabolism drawing energy from reactions of basic molecules such as H_2_, CO_2_, NH_3_, H_2_S, and phosphate (Wimmer et al.). This is further supported by the analysis of the phosphorus redox cycle, which had a significant role in the bioenergetics of the early evolution of life. Because reduced phosphorus compounds, such as phosphide minerals, have significant bioavailability, they could have provided energetic impetus during the first steps of early life and the evolutionary process (Nicholls et al.).

Indeed, life and the maintenance of cells require energy to support cellular processes. In fact, the claim that chemical processes transform energy into cellular life becomes invalid if the genetic code of the organism guides current cellular processes. The simplest cell known to function and work would require a minimum of 452 genes to survive under laboratory conditions (Hutchison et al., 2016), which is very different from a number of theories except for a generic first scenario that focuses on polymers carrying genetic information (Hernández). According to the existing publications, there is a diversity of opinions about the origin of life. Indeed, we are aware that basic chemical elements comprise the functioning of all existing organisms. Nevertheless, the question, “Who was LUCA?” still needs an answer. However, we should have clear its conceptual minimum because LUCA must have a basic cellular machinery for survival and the required informational tools and molecules to support this functional activity (Hutchison et al., 2016). Interestingly, “The RNA World Hypothesis” (Robertson and Joyce, 2012) and the discovery of catalytic RNA have challenged some expectations, suggesting that life could have started without a standard genetic code and catalysts based on DNA and proteins.

Like pre-biotic RNA or early RNA, tRNAs found in three related bacterial phyla—Planctomycetes, Verrucomicrobia, and Chlamydia—have been detected in other domains of currently existing life, such as plants, animals and fungi. This supports the evolutionary theory of relatedness and suggests that primitive RNA may have served as the genetic material of LUCA (Rekadwad et al., 2023) before being replaced by DNA and may represent a major stepping stone to solving the enigma of the origin of life on Earth. Furthermore, over 4.5 billion years of evolution have seen infinite genetic variation and trial-and-error attempts. Predicting all the genetic changes that have occurred through the evolution of life on our planet is an impossible task. However, the current phylogenetic divergence between Bacteria and Archaea-Eukarya (Gribaldo and Brochier-Armanet, 2019) can evaluate these changes, potentially providing a solution to identify LUCA. In this context, Cuecas et al. have made an attempt to analyze the phylogeny of several taxa and suggest that anammox (anaerobic ammonium oxidation) bacteria show a fairly slow progression over their long evolutionary lifetime (ca. 2.5 billion years) and maintain the uniqueness of their genomes. Thus, the evolutionary fate of anammox could show a highly conserved genomic pattern over time representing a singular functional group with only a very limited amount of change observed throughout the evolutionary history of these bacteria, which represent a singular functional group. This is likely due to its adaptation to highly specific extreme habitats and resources (Cuecas et al.).

Currently, with the development of new-generation sequencing platforms and massive computational analysis, genetic studies of metagenome-assembled genomes (MAGs) from microbial dark matter (MDM) have tried to decipher the secrets of microbial life through various analyses. Some studies look at whole genomes of cultured microorganisms (Rekadwad et al., 2023), others examine uncultured microbial living forms (Wang and Feng) showing that SAGs are more reliable than MAGs for the detailed study of MDM. The search for uncultured and rarely cultured microorganisms can provide valuable information on microbial evolution and the discovery of novel, yet-to-be-discovered, bioactive compounds (Gattoni et al.).

Thus, future perspectives for understanding LUCA and the origin of life on Earth would involve the analysis of cellular processes that may be governed by unique genetic codes. Thus, studies of extremophiles and the exploitation of extremophilic niches may be key to uncovering secret lineages and evolutionary branches within the tree of life. Therefore, multiple disciplines are involved in current advancements and these diverse approaches are likely to provide an accelerated understanding of life on Earth and its evolutionary history.

## Author contributions

BR: Writing – review & editing, Writing – original draft. JG: Writing – review & editing, Writing – original draft. W-JL: Writing – review & editing, Writing – original draft.
